# Evaluation of a simultaneous adsorption device for cytokines and platelet–neutrophil complexes in vitro and in a rabbit acute lung injury model

**DOI:** 10.1186/s40635-021-00414-7

**Published:** 2021-09-27

**Authors:** Yumiko Sekiya, Kaoru Shimada, Hiroshi Takahashi, Chisa Kuga, Shunsuke Komachi, Keishi Miwa, Toru Kotani

**Affiliations:** 1 Medical Devices & Materials Research Lab., Advanced Materials Research Labs., Toray Industries, Inc., Shiga, Japan; 2 Department of Intensive Care Medicine, Showa University School of Medicine, Tokyo, Japan

**Keywords:** Platelet–neutrophil complex, Cytokine, Blood purification, ARDS

## Abstract

**Background:**

Platelet–neutrophil complexes (PNCs) readily migrate into tissues and induce tissue damage via cytokine or other pathogenic factors release. These actions are involved in onset and progression of acute respiratory distress syndrome (ARDS). Thus, simultaneous removal of cytokines and activated neutrophils, including PNCs by blood purification may prevent development of ARDS and enhance drug effects. The goal of this study was to examine the effect of a newly developed adsorption column (NOA-001) that eliminates cytokines and activated neutrophils in a lung injury model.

**Results:**

Adsorption of cytokines, such as IL-8, IL-6 and HMGB-1, and PNCs was first measured in vitro. Lung injury was induced by HCl and lipopolysaccharide intratracheal infusion in rabbits ventilated at a low tidal volume (7–8 mL/kg) and PEEP (2.5 cmH_2_O) for lung protection. Arterial blood gas, hematologic values, plasma IL-8, blood pressure and heart rate were measured, and lung damage was evaluated histopathologically in animals treated with 8-h direct hemoperfusion with or without use of NOA-001. The in vitro adsorption rates for IL-8, IL-6, HMGB-1, activated granulocytes and PNCs were 99.5 (99.4–99.5)%, 63.9 (63.4–63.9)%, 57.6 (57.4–62.1)%, 9.9 (-4.4–21.3)% and 60.9 (49.0–67.6)%, respectively. Absorption of PNCs onto fibers was confirmed microscopically. These adsorption effects were associated with several improvements in the rabbit model. In respiratory function, the PaO_2_/FIO_2_ ratios at 8 h were 314 ± 55 mmHg in the NOA-001 group and 134 ± 41 mmHg in the sham group. The oxygenation index and PaCO_2_ at 8 h were 9.6 ± 3.1 and 57.0 ± 9.6 mmHg in the sham group and 3.0 ± 0.8 and 40.4 ± 4.5 mmHg in the NOA-001 group, respectively (*p* < 0.05). Blood pH at 8 h reached 7.18 ± 0.06 in the sham group, but was maintained at 7.36 ± 0.03 (within the normal range) in the NOA-001 group (*p* < 0.05). In lung histopathology, fewer hyaline membrane and inflammatory cells were observed in the NOA-001 group.

**Conclusion:**

A column for simultaneous removal of cytokines and PNCs showed efficacy for improvement of pulmonary function in an animal model. This column may be effective in support of treatment of ARDS.

**Supplementary Information:**

The online version contains supplementary material available at 10.1186/s40635-021-00414-7.

## Background

Acute respiratory distress syndrome (ARDS) is a life-threatening disease defined as non-cardiogenic pulmonary edema, respiratory distress, and hypoxemia with a high mortality in critically ill patients and results from processes that directly or indirectly compromise the lung [1]. Therapies for ARDS [2] include respiratory support using a ventilator and treatment with steroids, anticoagulants, and a neutrophil elastase inhibitor [3]. However, the efficacy of these methods is insufficient and improved strategies are required.

The pathophysiological mechanism leading to ARDS is complex, but it is clear that it includes an uncontrolled inflammatory response to a local or systemic insult. Cytokines such as interleukin (IL)-8, IL-6 and high-mobility group box 1 protein (HMGB-1) released from resident lung cells or blood cells in response to a harmful mediator and neutrophils activated by these cytokines are involved in onset and progression of ARDS [4, 5]. Cytokines and activated neutrophils are observed in patients with ARDS and blocking of these agents suppresses exacerbation of respiratory function in animal models [5–7].

Activated neutrophils have recently been shown to interact with platelets and form so-called platelet–neutrophil complexes (PNCs). Compared with neutrophils alone, these complexes readily adhere to the endothelium via upregulation of CD11b/CD18 (Mac-1) on neutrophils in a platelet P-selectin-dependent manner or through an interaction between adhesion molecules on platelets and the endothelium. Increased endothelial injury and permeability caused by proteases, reactive oxygen species, and leukotrienes from platelets or neutrophils and the increased migratory ability of neutrophils induced by chemotactic factors from platelets and the endothelium facilitate migration of PNCs into lung interstitium and alveolar spaces, in which the PNCs then release cytotoxic mediators [8, 9]. Formation of PNCs has also been associated with development of neutrophil extracellular traps (NETs), which are webs of extracellular DNA, histones, and proteolytic and bactericidal proteins from neutrophil granules. NETs function as a host defense system against microorganisms and also work as tissue injury mediators by regulating the innate immune response [10]. In patients with ARDS, PNCs and NETs may be correlated with severity of respiratory dysfunction [11–13], and for all the above reasons, PNCs are thought to be heavily involved in progression of ARDS.

Many clinical trials of ARDS treatment have been conducted over the last few decades, but most have failed because of insufficient efficacy [3]. We speculated that targeting of only one molecule may be limited for prevention of overall disease progression, because inflammatory cells and many cytokines repeatedly invade the lung in a complex manner during treatment and interfere with drug efficacy. Based on this hypothesis, inhibition of systemic inflammation by simultaneous adsorption of inflammatory cytokines and activated neutrophils, including PNCs, to reduce lung invasion may be effective and supportive treatment for ARDS. However, there is currently no medical device for such simultaneous adsorption, although several devices that adsorb either cytokines or granulocytes are used in clinical practice.

To test this hypothesis, we developed an extracorporeal blood purification column for simultaneous elimination of cytokines and activated neutrophils. We have previously described an apheresis device (CYT 860) that can eliminate several cytokines and bacterial exotoxins, which are so-called superantigens produced in Gram-positive bacteria. The device significantly reduced blood IL-6, IL-8 and IL-10 levels and improved the PaO_2_/FIO_2_ (P/F) ratio in seven patients with systemic inflammatory response syndrome in a clinical trial [14]. This column was redesigned to eliminate activated neutrophils simultaneously with cytokine adsorption. To our knowledge, our device is the first column to allow simultaneous adsorption of cytokines and PNCs. In the current study, we evaluated the effect of this new column (NOA-001) in vitro and in a rabbit model of acute lung injury. Mechanical ventilation under inappropriate conditions causes ventilator-induced lung injury by alveolar overdistention and/or cyclic collapse and reopening [15]. Thus, a lung-protective ventilation model was established using a low tidal volume and positive end-expiratory pressure (PEEP) for evaluation of the efficacy of the NOA-001 column for removal of cytokines and activated neutrophils, since it is important to avoid overlap of mechanical lung damage with inflammatory damage.

## Materials and methods

### Preparation of the adsorption column

A polystyrene-based composite fiber reinforced with polypropylene was prepared and chemically modified as described previously [16]. Briefly, the fiber was treated with *N*-methylol-α-chloroacetamide in a mixture of sulfuric acid and nitrobenzene on ice for 2 h in the presence of paraformaldehyde; then reacted with tetraethylenepentamine in dimethyl sulfoxide (DMSO) for 3 h at 40 °C; and then with 4-chlorophenylisocyanate in DMSO for 1 h at 30 °C. After thorough washing with DMSO followed by pyrogen-free water, the column was packed with 11.0 cm^3^ knitted fibers of size about 1/13th of the planned clinical size and filled with pyrogen-free saline. The priming volume was about 8 mL. The column was then sterilized using gamma ray irradiation (25,000 Gy).

### In vitro adsorption of cytokines

Chemically modified fiber of 0.055 cm^3^ was incubated with 2000 pg/mL IL-8, 2000 pg/mL IL-6 and 100 ng/mL HMGB-1 in 1.65 mL fetal bovine serum (FBS) at 37 °C for 2 h with gentle rotation. Cytokine-containing FBS without the fiber was incubated at 37 °C for 2 h as a blank sample. Concentrations of IL-8 and IL-6 in the supernatant were measured using ELISA (R & D Systems, Minneapolis, MN, USA). HMGB-1 was measured by Shino-Test Corp. (Tokyo, Japan). The adsorption ratio (%) was calculated as [(concentration of blank sample – concentration of test sample)/concentration of blank sample] × 100.

### In vitro adsorption of activated inflammatory cells

Lipopolysaccharide (LPS) (*E. coli* O55-B5, Sigma-Aldrich Co. LLC, St. Louis, MO, USA) was added at 70 EU/mL (11 ng/mL in used lot) to heparinized human whole blood samples from a healthy volunteer. After 30 min incubation, the blood was passed at a flow rate of 0.63 mL/min through the adsorption column filled with 0.86 cm^3^ of fiber. Blood samples were collected at the column inlet and outlet. Activated granulocytes and PNCs were analyzed by flow cytometry. We chose to use anti-CD62P antibody to identify PNCs that are actually involved in progression of tissue injury, because such PNCs are formed via an interaction between CD62P on platelets and PSGL-1 on neutrophils and some of PNCs’ functions are CD62P dependent [17, 18]. Blood samples were incubated for 15 min at r.t. with APC-anti CD11b (activated) (CBRM1/5, Biolegend, San Diego, CA, USA), BV421-anti-CD62P (AK4, Biolegend), BV510-anti-CD45 (HI30, Biolegend) and FITC-anti CD66b (G10F5, BD Biosciences, Franklin Lakes, NJ, USA). Other blood samples were incubated with isotype controls (Biolegend or BD Biosciences). Red blood cells were lysed and cells were rinsed using PBS(−) and resuspended in buffer. After adding 7-AAD reagent (Biolegend) and Flow-Count (Beckman Coulter, Brea, CA, USA), cells were characterized by BD FACSCanto II (BD Biosciences) and data were analyzed using FlowJo (FlowJo, LLC., Ashland, OR, USA). Neutrophils, the most abundant type of granulocytes, were identified as CD45^+^/CD66b^+^ cells, and PNCs were identified as CD11b (activated)^+^/CD62P^+^ cells among the neutrophils. Because almost all the cells in LPS-treated blood were CD11b (activated)^+^, blood without LPS was also processed in the same way to show elimination of CD11b (activated)^−^ cells. The absolute number of cells was calculated using Flow-Count. The adsorption ratio (%) was calculated as [(absolute cell number of inlet sample – absolute cell number of outlet sample)/absolute cell number of inlet sample] × 100. For histopathological observation of neutrophils and PNCs, the fiber after contact with blood was fixed in 50% Karnovsky fixative and osmic acid. After imbedding in resin, fiber was sectioned and stained with toluidine blue.

### Animal model with lung-protective ventilation

Male New Zealand white rabbits (2.8–3.4 kg) were obtained from Kitayama Labes (Nagano, JP) and fed with a standard diet and water ad libitum. Rabbits were anesthetized by intravenous administration of sodium pentobarbital (30 mg/kg) and anesthesia was maintained by continuous infusion of sodium pentobarbital (8.2 mg/kg/h) and vecuronium bromide (0.06 mg/kg/h) through the jugular vein. After placement in the supine position and local anesthesia with 0.5% lidocaine, tracheotomy was performed and a 16 Fr endotracheal tube was intubated. Rabbits were ventilated using a mechanical ventilator (EVITA300, Drager, Lübeck, DE) in VC-AC mode with FIO_2_ of 1.0 and PEEP of 2.5 cmH_2_O. After alveolar recruitment, the respiration rate was adjusted to maintain a tidal volume of 7–8 mL/kg to achieve a blood pH of 7.35–7.45 at baseline. The animals were maintained at a constant body fluid balance by continuous infusion of physiological saline at 2 mL/kg/h from the jugular vein. The carotid artery was cannulated for monitoring arterial blood pressure and heart rate (HR) and for sampling of arterial blood for blood gas analysis, hematology and cytokine measurement. Lung injury was induced by intratracheal (i.t.) administration of 0.04 N HCl followed by 0.05 mg/kg LPS (*E. coli* O55-B5, Sigma-Aldrich) after a 30 min interval. In the 2 h after LPS administration, arterial blood pH was adjusted and maintained at 7.35 to 7.45 by changing the respiration rate. If end-inspiratory pause was observed, the inspiratory time was adjusted throughout the experiment.

### Direct hemoperfusion

Rabbits were randomized in two groups after lung injury: the NOA-001 treated group (*n* = 7) and a sham group (*n* = 6) treated with an empty column with the same priming volume as that of NOA-001. The column was connected to the circuit and rinsed with heparinized saline. Unfractionated heparin was injected into the circuit at 60 IU/kg at the beginning of hemoperfusion, and continuously infused at 30–60 IU/kg/h from the anticoagulant injection port. The time of LPS administration was considered to be 0 h. Direct hemoperfusion was started at 15 min and continued for 8 h after LPS administration. Blood access was from the femoral artery to femoral vein and the flow rate was maintained at 5 mL/min using a peristaltic pump. The priming volume of the column with the circuit was 20 mL. Blood gas parameters (PaO_2_, PaCO_2_, pH and lactate) were measured using an i-STAT 200 portable clinical analyzer (Abbott, Chicago, IL, USA) and i-STAT cartridge CG4+ (Abbott) immediately after blood sampling. Hematological parameters (neutrophils, platelets) were measured using an XT-2000i analyzer (Sysmex, Hyogo, JP). Arterial blood pressure and HR were recorded and mean arterial pressure (MAP) was calculated using PowerLab systems (PowerLab8/30, LabChart7, AD Instruments, Sydney, Australia). Ventilation parameters were automatically saved in the ventilator. The oxygenation index (OI) was calculated as mean airway pressure/P/F ratio × 100. The plasma concentration of IL-8 was measured using an ELISA for rabbit IL-8 (Raybio, Norcross, GA, USA), and that of LPS was measured using Limulus ES-II Single Test Wako (Wako, Osaka, JP) and Toxinometer ET-5000 (Wako).

### Histopathological analysis

Rabbits were euthanized by exsanguination under anesthesia with pentobarbital at 8 h. The whole lung was removed and inflated with 10% formaldehyde neutral buffer solution to a pressure of 20 cmH_2_O via the trachea, and then fixed in the same buffer solution. After fixation, the lung was divided into 4 sections and each section was stained with hematoxylin–eosin (HE).

### Statistical analyses

Data are presented as the median and IQR for in vitro studies and the mean ± SEM for in vivo studies. Two rabbits in the NOA-001 group and two in the sham group died between 7 and 8 h. The 8 h values of one rabbit in the NOA-001 group were treated as defective data, because we were unable to collect a blood sample just before death. The 8 h values of the other three rabbits were substituted by the value measured just before death. Statistical analysis was performed using GraphPad Prism v.8.2.0 (GraphPad Prism Software, Inc., San Diego, CA). For in vitro adsorption of activated inflammatory cells, data were analyzed by Kruskal–Wallis test. In the in vivo study, baseline values in the NOA-001 and sham groups were compared by Mann–Whitney *U* test. Blood gas parameters, plasma IL-8, blood neutrophil counts, MAP, HR and plasma LPS in the two groups were compared by two way ANOVA with a Sidak post hoc test or Tukey post hoc test. Inlet and outlet levels of plasma IL-8 and blood neutrophils were compared by Wilcoxon signed-rank test.

## Results

### In vitro adsorption of cytokines and activated inflammatory cells

**Fig. 1:**
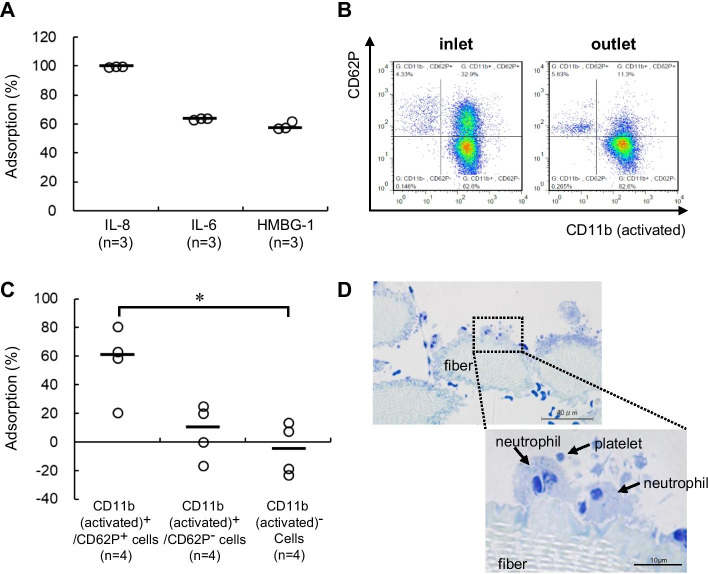
In vitro adsorption of cytokines and inflammatory cells. **a** Adsorption rates of IL-8, IL-6 and HMGB-1. **b** Representative scatter plot at the inlet and outlet of the column in flow cytometry. **c** Adsorption rate calculated from flow cytometric analysis. **d** Toluidine blue staining of fiber after contact with activated human blood. Arrows indicate NPC. Data are shown as medians (*n* = 3 or 4) and an open circle indicates the individual value. *p < 0.05 for CD11b (activated)^+^/CD62P^+^ cells vs. CD11b (activated)^−^ cells by Kruskal–Wallis test

Adsorption by the NOA-001 column was examined in vitro by incubating 0.055 cm^3^ of fiber with three cytokines (2000 pg/mL IL-8, 2000 pg/mL IL-6 and 100 ng/mL HMGB-1) in 1.65 mL of FBS at 37 °C for 2 h. The adsorption rates were 99.5 (99.4–99.5)%, 63.9 (63.4–63.9)% and 57.6 (57.4–62.1)% for IL-8, IL-6 and HMGB-1, respectively (Fig. 1a). As shown in Fig. 1b, c, CD11b (activated)^+^/CD62P^+^ cells and CD11b (activated)^+^/CD62P^−^ cells were eliminated by NOA-001, with adsorption rates of 60.9 (49.0–67.6)% and 9.9 (− 4.4 to 21.3)%, respectively. In contrast, the adsorption rate was − 5.6 (− 19.8 to 9.0)% for CD11b (activated)^−^ cells, indicating that these cells were not eliminated (Fig. 1c). There was a significant difference in elimination of CD11b (activated)^+^/CD62P^+^ cells and CD11b (activated)^−^ cells. Observation of the fibers after the adsorption test using an optical microscope showed adhesion of neutrophils and platelets that formed PNCs on the surface (Fig. 1d).

### Blood purification in a rabbit acute lung injury model with lung-protective ventilation

Prior to use of the acute lung injury model, we assessed the effect of mechanical ventilation using healthy rabbits treated with empty column. During 12-h ventilation with a tidal volume of 7–8 mL/kg and PEEP of 2.5 cmH_2_O, respiratory functions (P/F ratio, PaCO_2_, arterial blood pH and respiratory system compliance) did not change, indicating that ventilator-induced lung injury did not occur under these conditions (Additional file 2). Therefore, these settings for low tidal volume and PEEP were used for lung-protective ventilation in the following experiments.

As shown in Table 1, there were no differences between the NOA-001 and sham groups in baseline plasma IL-8 levels, blood neutrophil counts, blood gas parameters and hemodynamic parameters. Fifteen minutes after lung injury (just before starting hemoperfusion), there were also no differences in blood gas parameters, plasma IL-8 level and blood neutrophils. MAP before lung injury and HR at 15 min were significantly lower in the NOA-001 group, but these values recovered at 30 min and no significant differences were observed.

**Table 1:** Baseline characteristics

Variable	Before lung injury		After 15 min	
	Sham	NOA-001	Sham	NOA-001
Plasma IL-8, pg/mL	11.0 ± 1.5	9.4 ± 0.8	9.6 ± 0.8	9.6 ± 0.8
Neutrophils, × 10^2^ cells/μL	18.6 ± 2.9	18.9 ± 1.7	23.8 ± 2.7	21.0 ± 0.5
P/F, mmHg	530 ± 28	535 ± 21	303 ± 55	315 ± 38
pH	7.41 ± 0.01	7.41 ± 0.01	7.35 ± 0.02	7.38 ± 0.01
Lactate, mmol/L	1.05 ± 0.33	0.97 ± 0.17	0.84 ± 0.18	0.84 ± 0.11
MAP, mmHg	97.0 ± 3.5	89.3 ± 2.0 *	94.9 ± 5.0	86.4 ± 2.0
HR, beats/min	322 ± 11	307 ± 8	316 ± 7	291 ± 5*

*p < 0.05 for sham vs. NOA-001 groups by Mann–Whitney *U* test

**Fig. 2:**
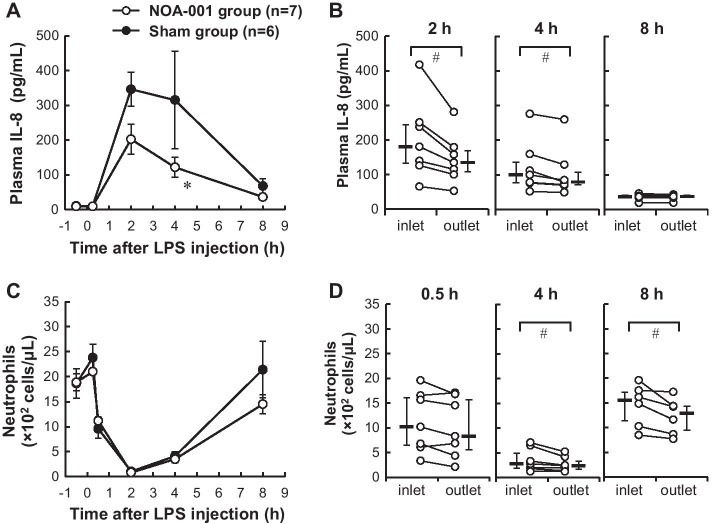
Changes in **a** plasma IL-8 and **b** plasma IL-8 at the inlet and outlet of the NOA-001 column. Each symbol indicates an individual value. **c** Blood neutrophil counts. **d** Blood neutrophil counts at the inlet and outlet. Each symbol indicates an individual value (**b** and **d**). Data are shown as mean ± SEM (**a** and **c**) and median and IQR (**b** and **d**). *p < 0.05 for NOA-001 vs. sham by two way ANOVA with a Sidak post hoc test. ^#^*p* < 0.05 for inlet vs. outlet by Wilcoxon signed-rank test

Plasma IL-8 concentrations transiently increased after HCl and LPS i.t. administration, and reached a peak concentration at 2 h in both groups (Fig. 2a). At 4 h, the IL-8 level was significantly lower in the NOA-001 group compared with the sham group (121.9 ± 28.8 vs. 315.3 ± 139.7 pg/mL, *p* < 0.05). In addition, IL-8 decreased in the column and the levels at the column outlet were significantly lower than those at the inlet at 2 h (145.8 ± 27.3 vs. 202.6 ± 43.3 pg/mL, *p* < 0.05) and 4 h (106.0 ± 27.3 vs. 121.9 ± 28.8 pg/mL, *p* < 0.05) (Fig. 2b).

Changes in blood neutrophil counts are shown in Fig. 2c. In both groups, a rapid and transient decrease was observed after LPS administration and the neutrophil count reached a minimum at 2 h. Neutrophils subsequently increased and counts in the NOA-001 group tended to be lower than those in the sham group, but without a significant difference. Neutrophil counts at the column outlet were significantly lower than those at the inlet at 4 h (2.7 ± 0.3 × 10^2^ vs. 3.5 ± 0.7 × 10^2^ cells/μL, *p* < 0.05) and 8 h (12.3 ± 1.6 × 10^2^ vs. 14.5 ± 1.9 × 10^2^ cells/μL, *p* < 0.05) (Fig. 2d).

**Fig. 3:**
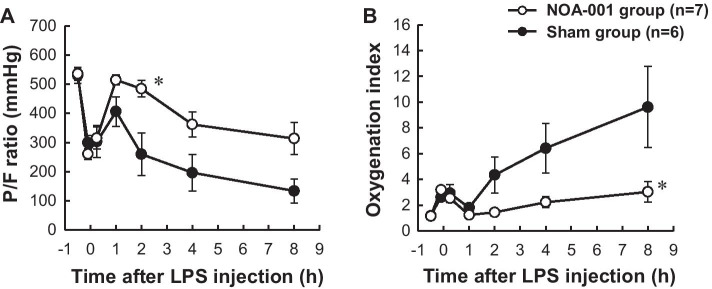
Changes in pulmonary gas exchange parameters. **a** *P*/*F* ratio and **b** oxygenation index. Data are shown as mean ± SEM. *p < 0.05 for NOA-001 vs. sham by two way ANOVA with a Tukey post hoc test

Immediately after HCl and LPS i.t. administration, P/F ratios decreased transiently and then recovered within 1 h in both groups. In the sham group, the P/F ratio subsequently decreased and reached 134 ± 41 mmHg at 8 h (Fig. 3a). In contrast, in the NOA-001 group, the P/F ratio was maintained above 300 mmHg until 8 h, with a significant difference compared to the sham group at 2 h (484 ± 28 vs. 260 ± 73 mmHg, *p* < 0.05). PaCO_2_ transiently increased and then recovered within 2 h in both groups, but then gradually increased in the sham group. In contrast, in the NOA-001 group, PaCO_2_ was maintained up to 8 h and there was a significant difference between the groups at 8 h (Additional file 1). Because the airway diameter is small in rabbits, we expected that airway pressure would affect gas exchange, and thus, the OI was also calculated. The OI increased in the 8 h period in both groups, but was significantly lower in the NOA-001 group compared to the sham group at 8 h (3.03 ± 0.81 vs. 9.62 ± 3.14, *p* < 0.05) (Fig. 3b).

**Fig. 4:**
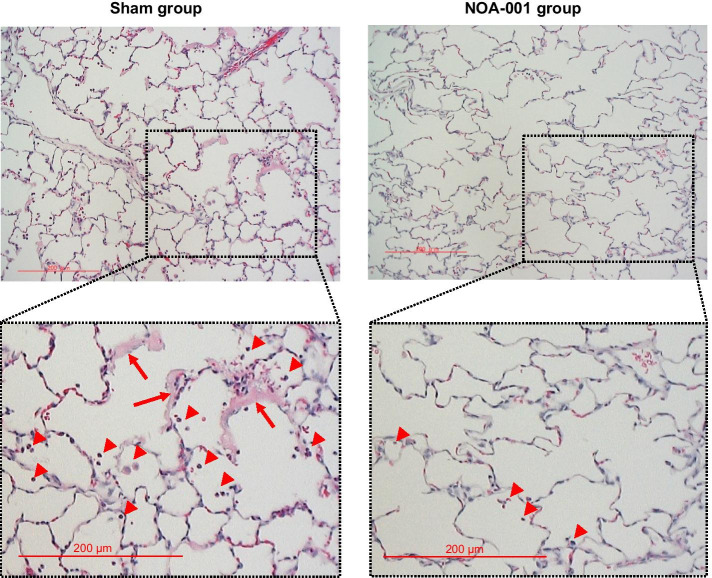
Histopathological observation of the lung with HE staining at 8 h. Red arrowheads indicate migrated inflammatory cells and red arrows indicate hyaline membranes. Scale bars represent 200 μm

Histopathological observation at 8 h (Fig. 4) revealed inflammatory cell migration in the alveolar space and hyaline membrane formation in the alveolar wall in the sham group. These findings are characteristics of the exudative phase of ARDS. In the NOA-001 group, counts of migrated inflammatory cells were low and hyaline membrane formation was not observed. Obvious differences for other findings, such as hemorrhage and alveolar wall thickness, were not detected between the two groups.

**Fig. 5:**
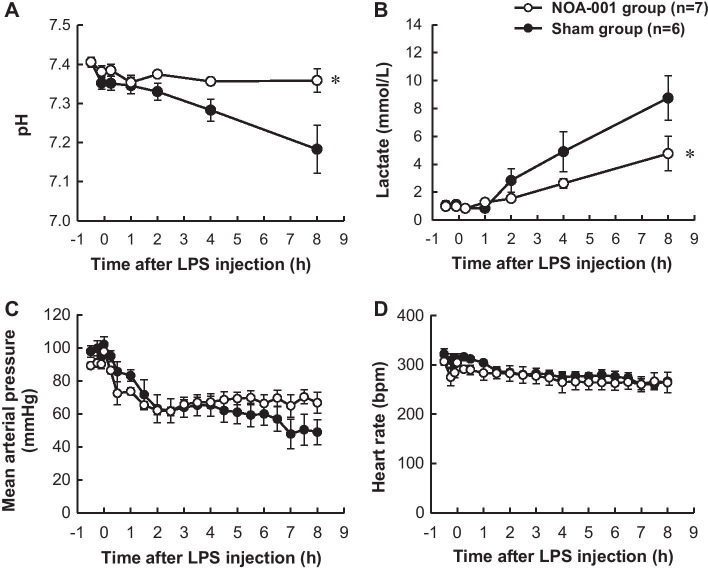
Changes in **a** arterial blood pH, **b** lactate levels, **c** mean arterial pressure and **d** heart rate. Data are shown as mean ± SEM. *p < 0.05 for NOA-001 vs. sham by two way ANOVA with a Sidak post hoc test or Tukey post hoc test

There was an increase in lactate and a decrease in arterial blood pH in the sham group, whereas arterial blood pH was unchanged and lactate was maintained at a lower level in the NOA-001 group (Fig. 5), with a significant difference at 8 h (Fig. 5a, b). In hemodynamic parameters, MAP gradually decreased until 2 h and then remained almost constant, with no significant difference between the two groups (Fig. 5c). HR in both groups remained in the range from 260 to 310 bpm throughout the experiment (Fig. 5d). Plasma LPS in the sham group transiently increased to 23.5 ± 10.1 pg/mL at 1 h and then decreased to 17.8 ± 13.7 pg/mL at 8 h. In the NOA-001 group, this level showed similar changes and was 13.8 ± 5.7 pg/mL at 1 h and 4.7 ± 3.4 pg/mL at 8 h. There was no significant difference between the groups (Additional file 1).

## Discussion

This study indicates that simultaneous removal of cytokines and PNCs using extracorporeal blood purification results in decreased blood cytokine concentrations and decreased migration of neutrophils in lung tissue, followed by improvement in respiratory functions and acidosis within 8 h. These results suggest that the NOA-001 column intercepted activated neutrophils including PNCs and that continuous removal of cytokines prevented reactivation of neutrophils and tissue invasion over a short period. In addition, in another study, NOA-001 was treated for 24 h in healthy rabbits to examine the biocompatibility. Although decrease in blood platelet count by about 60% was observed, there were not any other serious effects (Additional file 3).

In treatment of hyperinflammatory diseases, such as ARDS or sepsis, suppression of systemic inflammation by devices or drugs is effective in the early stage. Steroids are the major treatment for this hyperinflammation; however, steroids work at the infection focus and have a side effect of immunosuppression that may become a problem [19]. In contrast, blood purification affects only the circulating blood and does not act directly on the infection focus. General side effects of a hemoadsorption column include adsorption of serum substances, bleeding due to excessive use of an anticoagulant, and complications associated with catheter insertion, but these can be avoided by careful monitoring of patients.

Cytokine removal with a hemoadsorption column avoids side effects and suppresses systemic inflammation, and can be used to treat sepsis, ARDS and COVID-19 [20, 21]. These studies suggest the efficacy of improvement of gas exchange or organ failure by removal of cytokines from circulating blood; however, some reports have indicated that the efficacy of hemoadsorption devices is insufficient for an improvement in outcome [22].

We speculated that previously used hemoadsorption devices have removed only cytokines and are not enough to suppress activation of neutrophils and formation of PNCs that produce cytokines and induce tissue damage. Thus, these devices cannot suppress inflammation completely in the early stage of the disease. Activated neutrophils or NETs are involved in onset and progression of ARDS [4]. In addition, circulating NPC levels are significantly higher in COVID-19 patients than in healthy donors [13] and activated neutrophils may play a central role in the pathogenesis of severe COVID-19 [23]. Therefore, it is likely that rapid removal of both cytokines and activated neutrophils including PNCs simultaneously will be more effective for treatment of the early stage of these diseases.

Our in vitro study indicated that the NOA-001 column can adsorb cytokines and activated neutrophils including PNCs. The mechanism of adsorption involves phagocytosis and selective adsorption of activated neutrophils. This selectivity may minimize unfavorable immunosuppressive effects by adsorption of unactivated neutrophils. We then used the NOA-001 column for treatment of a rabbit model of acute lung injury induced by administration of HCl and LPS i.t. Acid aspiration or LPS instillation have each been reported to induce PNCs in mouse [8, 24]. Therefore, we used two-hit injury with both HCl and LPS to induce PNCs and reflect multiple injury hits in clinical patients. To observe the efficacy of removal of cytokines and PNCs, ventilator-induced lung injury was prevented by lung-protective ventilation (7–8 mL/kg tidal volume and 2.5 cmH_2_O PEEP) [25]. Damage to the alveolar walls was slight, which indicated that ventilation did not damage the lung tissue and that this model can be used to evaluate effects on inflammation damage only.

Decreases of IL-8 and activated neutrophils were associated with suppression of lung function, such as P/F ratio and OI. The histopathological observation that hyaline membrane formation and inflammatory cell migration were suppressed suggested that vascular and alveolar leakage was avoided by removal of cytokines and activated neutrophils in the exudative phase of acute lung injury. However, slight inflammatory cell migration was observed after NOA-001 treatment. Intratracheal administration of LPS can induce migration of polymorphonuclear leukocytes to lung tissue [26], and neutrophils may have been activated and migrated to lung tissue immediately after direct i.t. administration of LPS, before elimination by NOA-001 treatment.

Significant differences in arterial blood pH and lactate levels were observed between the two groups, but the LPS concentration and MAP decreases did not differ significantly. Our clinical pilot study using CYT-860, a prototype of NOA-001, also showed a significant increase in P/F ratio, whereas changes in hemodynamic factors were slight and not significant [14]. These results indicate that hemodynamic changes are not the major reason for the differences in P/F ratio and OI. However, lactate improvement has also been reported in cytokine removal therapy in a clinical study [27, 28], which suggests that removal of inflammatory factors can affect lung and other peripheral vascular damage. Further animal studies or clinical studies in non-septic ARDS cases are needed to examine this possibility.

In this study, we used a HCl and LPS intratracheal administration model. Acid aspiration or LPS instillation induces PNCs in mice [8, 24], but mice and rats are too small for extracorporeal circulation. Therefore, we chose rabbit as the model animal. Rabbits are used in many studies, including for analysis of extracorporeal circulation and measurement of cytokines. The measurement of plasma IL-8 levels proved the capacity of NOA-001 to adsorb IL-8 during hemoperfusion. However, there are some limitations in the rabbit model, including the duration of observation. Anesthesia, paralysis and fixing of the rabbits in the supine position leads to gravitational atelectasis by itself and the number of deaths increased at more than 8-h observation in a preliminary lung injury study (data not shown). Therefore, we limited the study to 8 h and, due to this limitation, we used LPS for rapid induction of lung damage. However, the possible effect of LPS makes the results less clear. Moreover, the severity of lung injury was limited to the mild stage to keep the animal alive for 8 h in the lung protective ventilated rabbit injury model; therefore, lung injury assessed by histopathological analysis was mild, even in the sham group. Secondly, the rabbit lung is small and we were unable to perform additional tests to determine the degree of lung injury, such as permeability of vascular leakage and pulmonary edema. To address all of these issues, studies are needed in large animals in a standing position with use of analgesic, but without anesthesia, in a special facility, with measurement of permeability based on the wet-to-dry weight ratio or lymph flow measurement over a longer duration.

## Conclusion

A new adsorption column (NOA-001) was designed and developed for simultaneous elimination of cytokines and activated neutrophils with PNCs. The NOA-001 column suppressed deterioration of gas exchange in rabbits by eliminating inflammatory factors from circulating blood. Therefore, this column may be a new medical device for support of treatment of ARDS by preventing tissue damage by activated neutrophils and PNCs in early-stage lung injury. Further studies in different lung injury models are warranted to confirm the lung-protective mechanism of the NOA-001 column.

## Supplementary Information

**Additional file 1:** Measurement parameters in a rabbit acute lung injury model.

**Additional file 2:** Measurement parameters in ventilated healthy rabbits treated with empty column

**Additional file 3:** Measurement parameters in healthy rabbits treated with NOA-001.

## Data Availability

The data sets used and/or analyzed during the current study are available from the corresponding author on reasonable request.

## Abbreviations

ARDS: Acute respiratory distress syndrome
FBS: Fetal bovine serum
HE: Hematoxylin–eosin
HMGB-1: High-mobility group box 1
HR: Heart rate
IL: Interleukin
LPS: Lipopolysaccharide
i.t.: Intratracheal
MAP: Mean arterial pressure
NET: Neutrophil extracellular trap
NOA-001: Tested device
OI: Oxygenation index
PEEP: Positive end-expiratory pressure
P/F: PaO_2_/FIO_2_
PNC: Platelet-neutrophil complex

## References

[1] Niall D, Ferguson ND, Fan E, Camporota L, Antonelli M, Anzueto A, Beale R, Brochard L, Brower R, Esteban A, Gattinoni L, Rhodes A, Slutsky AS, Vincent JL, Rubenfeld GD, Thompson BT, Ranieri VM (2012). The Berlin definition of ARDS: an expanded rationale, justification, and supplementary material. Intensive Care Med.

[2] The Faculty of Intensive Care Medicine, the Intensive Care Society (2018) Guideline of the management of acute respiratory distress syndrome.

[3] Gonzaleza H, Horiea S, Laffey JG (2021). Emerging cellular and pharmacologic therapies for acute respiratory distress syndrome. Curr Opin Crit Care.

[4] Matthay MA, Zemans RL, Zimmerman GA, Arabi YM, Beitler JR, Mercat A, Herridge M, Randolph AG, Calfee CS (2019). Acute respiratory distress syndrome. Nat Rev Dis Primers.

[5] Blondonnet R, Constantin JM, Sapin V, Jabaudon M (2016). A pathophysiologic approach to biomarkers in acute respiratory distress syndrome. Dis Markers.

[6] Takala A, Jousela I, Takkunen O, Kautiainen H, Jansson SE, Orpana A, Karonen SI, Repo H (2002). A prospective study of inflammation markers in patients at risk of indirect acute lung injury. Sock.

[7] Hoth JJ, Wells JD, Hiltbold EM, McCall CE, Yoza BK (2011). Mechanism of neutrophil recruitment to the lung after pulmonary contusion. Shock.

[8] Zarbock A, Singbartl K, Ley K (2006). Complete reversal of acid-induced acute lung injury by blocking of platelet-neutrophil aggregation. J Clin Invest.

[9] Page C, Pitchford S (2013). Neutrophil and platelet complexes and their relevance to neutrophil recruitment and activation. Int Immunopharmacol.

[10] Kim SK, Jenne CN (2016). Role of platelets in neutrophil extracellular trap (NET) production and tissue injury. Semin Immunol.

[11] Lefrançais E, Mallavia B, Zhuo H, Calfee CS, Looney MR (2018). Maladaptive role of neutrophil extracellular traps in pathogen-induced lung injury. JCI Insight.

[12] Li H, Zhou X, Tan H, Hu Y, Zhang L, Liu S, Dai M, Li Y, Li Q, Mao Z, Pan P, Su X, Hu C (2018). Neutrophil extracellular traps contribute to the pathogenesis of acid-aspiration-induced ALI/ARDS. Oncotarget.

[13] Manne BK, Denorme F, Middleton EA, Portier I, Rowley JW, Stubben C, Petrey AC, Tolley ND, Guo L, Cody M, Weyrich AS, Yost CC, Rondina MT, Campbell RA (2020). Platelet gene expression and function in patients with COVID-19. Blood.

[14] Kobe Y, Oda S, Matsuda K, Nakamura M, Hirasawa H (2007). Direct hemoperfision with cytokine-adsorbing device for the treatment of patient or severe hypercytokinemia: a pilot study. Blood Purif.

[15] Prost ND, Dreyfuss D (2012). How to prevent ventilator-induced lung injury?. Minerva Anestesiol.

[16] Miwa K, Fukuyama M, Ida N, Igarashi H, Uchiyama T (2003). Preparation of a superantigen-adsorbing device and its superantigen removal efficacies in vitro and in vivo. Int J Infect Dis.

[17] Peters MJ, Heyderman RS, Hatch DJ, Klein NJ (1997). Investigation of platelet-neutrophil interactions in whole blood by flow cytometry. J Immunol Methods.

[18] Peters MJ, Dixon G, Kotowicz KT, Hatch DJ, Heyderman RS, Klein NJ (1999). Circulating platelet-neutrophil complexes represent a subpopulation of activated neutrophils primed for adhesion, phagocytosis and intracellular killing. Br J Haematol.

[19] Caplan A, Fett N, Rosenbach M, Werth VP, Micheletti RG (2017). Prevention and management of glucocorticoid-induced side effects: a comprehensive review. J Am Acad Dermatol.

[20] Rampino T, Gregorini M, Perotti L, Ferrari F, Pattonieri EF, Grignano MA, Valente M, Garrone A, Islam T, Libetta C, Sepe V, Albertini R, Bruno R, Belliato M (2020). Hemoperfusion with CytoSorb as adjuvant therapy in critically ill patients with SARS-CoV2 pneumonia. Blood Purif.

[21] Villa G, Romagnoli S, Rosa SD, Greco M, Resta M, Montin DP, Prato F, Patera F, Ferrari F, Rotondo G, Ronco C (2020). Blood purification therapy with a hemodiafilter featuring enhanced adsorptive properties for cytokine removal in patients presenting COVID-19: a pilot study. Crit Care.

[22] Schädler D, Pausch C, Heise D, Meier-Hellmann A, Brederlau J, Weiler N, Marx G, Putensen C, Spies C, Jörres A, Quintel M, Engel C, Kellum JA, Kuhlmann MK (2017). The effect of a novel extracorporeal cytokine hemoadsorption device on IL-6 elimination in septic patients: a randomized controled trial. PLoS ONE.

[23] Meizlish ML, Pine AB, Bishai JD, Goshua G, Nadelmann ER, Simonov M, Chang CH, Zhang H, Shallow M, Bahel P, Owusu K, Yamamoto Y, Arora T, Atri DS, Patel A, Gbyli R, Kwan J, Won CH, Cruz CD, Price C, Koff J, King BA, Rinder HM, Wilson FP, Hwa J, Halene S, Damsky W, Dijk DV, Lee AI, Chun HJ (2021). A neutrophil activation signature predicts critical illness and mortality in COVID-19. Blood Adv.

[24] Ortiz-Muñoz G, Mallavia B, Bins A, Headley M, Krummel MF, Looney MR (2014). Aspirin-triggered 15-epi-lipoxin A4 regulates neutrophil-platelet aggregation and attenuates acute lung injury in mice. Blood.

[25] Ogawa EN, Ishizaka A, Tasaka S, Koh H, Ueno H, Amaya F, Ebina M, Yamada S, Funakoshi Y, Soejima J, Moriyama K, Kotani T, Hashimoto S, Morisaki H, Abraham E, Takeda J (2006). Contribution of high-mobility group box-1 to the development of ventilator-induced lung injury. Am J Respir Crit Care Med.

[26] Yamamoto T, Kajikawa O, Martin TR, Sharar SR, Harlan JM, Winn RK (1998). The role of leukocyte emigration and IL-8 on the development of lipopolysaccharide-induced lung injury in rabbits. J Immunol.

[27] Mehta Y, Mehta C, Kumar A, George JV, Gupta A, Nanda S, Kochhar G, Raizada A (2020). Experience with hemoadsorption (CytoSorb®) in the management of septic shock patients. World J Crit Care Med.

[28] Paul R, Sathe P, Kumar S, Prasad S, Aleem M, Sakhalvalkar P (2021). Multicentered prospective investigator initiated study to evaluate the clinical outcomes with extracorporeal cytokine adsorption device (Cytosorb®) in patients with sepsis and septic shock. World J Crit Care Med.

