# Supplementary material for: Evaluation of a simultaneous adsorption device for cytokines and platelet–neutrophil complexes in vitro and in a rabbit acute lung injury model
Source: Intensive Care Med Exp. 2021 Sep 27;9:49. doi: 10.1186/s40635-021-00414-7 (PMC8473513; doi:10.1186/s40635-021-00414-7)
Supplement: Supplementary file 1 — Additional file 1: Measurement parameters in a rabbit acute lung injury model. [file 40635_2021_414_MOESM1_ESM.docx]

Supplementary Data. Measurement parameters in a rabbit acute lung injury model

|  | Group | Time after LPS injection (h) | | | | | | | |
| --- | --- | --- | --- | --- | --- | --- | --- | --- | --- |
|  |  | -0.5 | -0.1 | 0.25 | 0.5 | 1 | 2 | 4 | 8 |
| Plasma IL-8,  pg/mL | Sham group | 11.0 ± 1.5 | － | 9.6 ± 0.8 | － | － | 346.3 ± 48.2 | 315.3 ± 139.7 | 67.8 ± 20.4 |
|  | NOA-001 group | 9.4 ± 0.8 | － | 9.6 ± 0.8 | － | － | 202.6 ± 43.3 | 121.9 ± 28.8* | 36.3 ± 3.2 |
| Neutrophils,  ×10^2^ cells/μL | Sham group | 18.6 ± 2.9 | － | 23.8 ± 2.7 | 9.6 ± 1.9 | － | 1.1 ± 0.1 | 4.1 ± 0.8 | 21.4 ± 5.6 |
|  | NOA-001 group | 18.9 ± 1.7 | － | 21.0 ± 0.5 | 11.2 ± 0.8 | － | 0.8 ± 0.1 | 3.5 ± 0.7 | 14.5 ± 1.9 |
| P/F, mmHg | Sham group | 530 ± 28 | 300 ± 24 | 303 ± 55 | － | 406 ± 50 | 260 ± 73 | 196 ± 62 | 134 ± 41 |
|  | NOA-001 group | 535 ± 21 | 261 ± 19 | 315 ± 38 | － | 514 ± 17 | 484 ± 28* | 362 ± 44 | 314 ± 55 |
| OI | Sham group | 1.17 ± 0.07 | 2.62 ± 0.26 | 2.92 ± 0.66 | － | 1.80 ± 0.32 | 4.34 ± 1.41 | 6.42 ± 1.93 | 9.62 ± 3.14 |
|  | NOA-001 group | 1.14 ± 0.05 | 3.19 ± 0.26 | 2.54 ± 0.34 | － | 1.24 ± 0.04 | 1.44 ± 0.11 | 2.23 ± 0.44 | 3.03 ± 0.81* |
| PaCO_2_, mmHg | Sham group | 48.5 ± 1.5 | 53.1 ± 2.4 | 54.7 ± 2.8 | － | 55.6 ± 2.7 | 51.7 ± 1.9 | 50.7 ± 1.7 | 57.0 ± 9.6 |
|  | NOA-001 group | 45.7 ± 1.7 | 48.2 ± 2.6 | 48.4 ± 2.2 | － | 50.9 ± 1.9 | 46.7 ± 1.1 | 44.4 ± 0.9 | 40.4 ± 4.5* |
| pH | Sham group | 7.41 ± 0.01 | 7.35 ± 0.02 | 7.35 ± 0.02 | － | 7.35 ± 0.02 | 7.33 ± 0.02 | 7.28 ± 0.03 | 7.18 ± 0.06 |
|  | NOA-001 group | 7.41 ± 0.01 | 7.38 ± 0.01 | 7.38 ± 0.01 | － | 7.35 ± 0.02 | 7.37 ± 0.01 | 7.36 ± 0.01 | 7.36 ± 0.03* |
| Lactate, mmol/L | Sham group | 1.05 ± 0.33 | 1.12 ± 0.25 | 0.84 ± 0.18 | － | 0.84 ± 0.04 | 2.84 ± 0.85 | 4.91 ± 1.43 | 8.75 ± 1.59 |
|  | NOA-001 group | 0.97 ± 0.17 | 0.97 ± 0.15 | 0.84 ± 0.11 | － | 1.27 ± 0.26 | 1.54 ± 0.07 | 2.63 ± 0.35 | 4.76 ± 1.23* |
| HCO_3_^-^, mmol/L | Sham group | 29.7 ± 0.7 | 28.7 ± 0.8 | 29.5 ± 0.5 | － | 30.0 ± 0.2 | 27.0 ± 0.9 | 23.9 ± 1.2 | 20.3 ± 1.5 |
|  | NOA-001 group | 28.1 ± 0.7 | 27.8 ± 0.7 | 28.3 ± 0.7 | － | 27.8 ± 0.7 | 26.9 ± 0.5 | 24.6 ± 0.7 | 22.2 ± 1.7 |
| B.E., mmol/L | Sham group | 5.5 ± 0.7 | 3.5 ± 0.8 | 4.3 ± 0.5 | － | 4.7 ± 0.2 | 1.2 ± 1.3 | -2.5 ± 1.6 | -7.8 ± 1.9 |
|  | NOA-001 group | 3.9 ± 0.7 | 3.3 ± 0.6 | 3.6 ± 0.8 | － | 2.6 ± 0.9 | 2.0 ± 0.4 | -0.7 ± 0.9 | -2.9 ± 1.4 |
| MAP, mmHg | Sham group | 97.9 ± 3.5 | 94.9 ± 5.0 | 95.1 ± 3.4 | 85.6 ± 6.0 | 83.2 ± 3.5 | 63.2 ± 8.4 | 65.4 ± 6.8 | 48.9 ± 7.5 |
|  | NOA-001 group | 89.3 ± 2.0 | 90.2 ± 2.9 | 86.4 ± 2.0 | 72.5 ± 7.1 | 73.7 ± 2.0 | 62.2 ± 2.6 | 67.1 ± 3.7 | 66.8 ± 6.3 |
| HR, bpm | Sham group | 322 ± 11 | 309 ± 11 | 316 ± 7 | 312 ± 6 | 304 ± 5 | 285 ± 6 | 274 ± 9 | 267 ± 5 |
|  | NOA-001 group | 307 ± 8 | 285 ± 11 | 291 ± 5 | 290 ± 10 | 284 ± 14 | 283 ± 16 | 265 ± 21 | 264 ± 21 |
| Plasma LPS,  pg/mL | Sham group | 1.1 ± 0.8 | － | － | － | 23.5 ± 10.1 | － | － | 17.8 ± 13.7 |
|  | NOA-001 group | 0.7 ± 0.6 | － | － | － | 13.8 ± 5.7 | － | － | 4.7 ± 3.4 |

*p<0.05 for NOA-001 vs. sham by two way ANOVA with a Sidak post hoc test or Tukey post hoc test.

－not measured
