# Supplementary material for: Evaluation of a simultaneous adsorption device for cytokines and platelet–neutrophil complexes in vitro and in a rabbit acute lung injury model
Source: Intensive Care Med Exp. 2021 Sep 27;9:49. doi: 10.1186/s40635-021-00414-7 (PMC8473513; doi:10.1186/s40635-021-00414-7)
Supplement: Supplementary file 2 — Additional file 2: Measurement parameters in ventilated healthy rabbits treated with empty column [file 40635_2021_414_MOESM2_ESM.docx]

Supplementary Data 2. Measurement parameters in ventilated healthy rabbits treated with empty column

|  | Time after vehicle injection (h) | | | | | | | |
| --- | --- | --- | --- | --- | --- | --- | --- | --- |
|  | -0.5 | 0.25 | 1 | 2 | 4 | 6 | 8 | 12 |
| P/F, mmHg | 588 | 578 | 602 | 569 | 607 | 616 | 614 | 598 |
| PaCO_2_, mmHg | 45.8 | 47.9 | 45.8 | 43.4 | 43.4 | 45.6 | 45.7 | 45.4 |
| pH | 7.43 | 7.43 | 7.43 | 7.44 | 7.45 | 7.44 | 7.43 | 7.40 |
| Compliance, mL/cmH2O | 4.26 | 4.62 | － | － | － | 2.45 | － | 2.75 |

Data are shown as mean of two rabbits.

－not measured
