# Supplementary material for: Evaluation of a simultaneous adsorption device for cytokines and platelet–neutrophil complexes in vitro and in a rabbit acute lung injury model
Source: Intensive Care Med Exp. 2021 Sep 27;9:49. doi: 10.1186/s40635-021-00414-7 (PMC8473513; doi:10.1186/s40635-021-00414-7)
Supplement: Supplementary file 3 — Additional file 3: Measurement parameters in healthy rabbits treated with NOA-001. [file 40635_2021_414_MOESM3_ESM.docx]

Supplementary Data 3. Measurement parameters in healthy rabbits treated with NOA-001

|  | Time after hemoperfusion start (h) | | | | | | | |  |
| --- | --- | --- | --- | --- | --- | --- | --- | --- | --- |
|  | 0 | 1 | 2 | 4 | 8 | 12 | 18 | 24 | |
| PaO_2_, mmHg | 92 ± 4 | 103 ± 8 | － | 98 ± 6 | 104 ± 5 | 108 ± 6 | 104 ± 5 | 103 ± 6 | |
| MAP, mmHg | 67.8 ± 6.0 | 57.4 ± 3.1 | 55.8 ± 5.9 | 59.1 ± 5.5 | 59.0 ± 5.4 | 55.0 ± 1.9 | 59.8 ± 3.9 | 57.3 ± 4.8 | |
| Platelets, ×10^3^ cells/μL | 341 ± 16 | 150 ± 23 | － | 167 ± 28 | 146 ± 17 | 177 ± 24 | 159 ± 36 | 174 ± 24 | |

Data are shown as mean ± SEM of four rabbits.

－not measured
